# Long-term effects of somatostatin analogues in rat GH-secreting pituitary tumor cell lines

**DOI:** 10.1007/s40618-021-01609-1

**Published:** 2021-06-14

**Authors:** A. Dicitore, D. Saronni, G. Gaudenzi, S. Carra, M. C. Cantone, M. O. Borghi, L. Persani, G. Vitale

**Affiliations:** 1 Laboratory of Geriatric and Oncologic Neuroendocrinology Research, Istituto Auxologico Italiano, IRCCS, Via Zucchi 18, 20095 Cusano Milanino, MI Italy; 2 Department of Medical Biotechnology and Translational Medicine, University of Milan, Milan, Italy; 3 Laboratory of Endocrine and Metabolic Research, Istituto Auxologico Italiano, IRCCS, Milan, Italy; 4 Experimental Laboratory of Immuno-rheumatology, Istituto Auxologico Italiano, IRCCS, Milan, Italy; 5 Department of Clinical Sciences and Community Health (DISCCO), University of Milan, Milan, Italy

**Keywords:** GH-secreting pituitary tumor, Acromegaly, Somatostatin analogs, Long-term treatment, Apoptosis

## Abstract

**Purpose:**

First-generation somatostatin analogs, octreotide (OCT) and lanreotide, are the cornerstone for the medical treatment of growth hormone (GH)-secreting pituitary tumors. A new multireceptor analog, such as pasireotide (PAS), showed better activity than OCT in long-term treatment of patients with acromegaly, but modulation of intracellular key processes is still unclear in vitro. In this study, we evaluated the antitumor activity of OCT and PAS in two GH-secreting pituitary tumor cell lines, GH3 and GH4C1, after a long-term incubation.

**Methods:**

The effects of PAS and OCT on the cell viability, cell cycle, apoptosis, GH secretion, and tumor-induced angiogenesis have been evaluated through a colorimetric method (MTS Assay), DNA flow cytometry with propidium iodide, and Annexin V-FITC/propidium iodide staining, ELISA assay and zebrafish platform, respectively.

**Results:**

PAS showed a more potent antitumor activity compared to OCT in GH3 cell line exerted through inhibition of cell viability, perturbation of cell cycle progression, and induction of apoptosis after 6 days of incubation. A concomitant decrease in GH secretion has been observed after 2 days of incubation only with PAS. No effect on tumor-induced angiogenesis has been reported after treatment with OCT or PAS in zebrafish/tumor xenograft model.

**Conclusion:**

Long-term incubation with PAS showed a more potent antitumor activity than that reported after OCT in GH3 cells, mainly modulated by a cell cycle perturbation and a relevant induction in apoptosis.

## Introduction

Growth hormone (GH)-secreting pituitary tumors account for about 30% of all functioning pituitary tumors. The excess of GH and insulin-like growth factor 1 (IGF-1) results in a disease known as acromegaly, that is associated with increased morbidity and mortality [1]. First line management for these patients is aimed at normalizing GH and IGF-1 levels, to ameliorate signs and symptoms of this disease and to reduce mortality [2].

Medical therapy is recommended for acromegalic patients who fail to achieve remission after surgery, and for patients who refuse or have contraindications to surgery. GH-secreting pituitary tumors predominantly express somatostatin receptor (SST) -2 and -5 [3]. Somatostatin receptor ligands (SRLs) selective for SST_2_, such as octreotide (OCT) and lanreotide, are the cornerstone for the medical therapy of these tumors [4, 5]. Long-term treatment of acromegaly with OCT and lanreotide has been widely studied and showed normalization of GH and IGF-1 levels in about 20–70% and tumor shrinkage in 36–75% of patients [6–9]. Therefore, a relevant group of patients showed partial or total resistance to SRLs [10]. This phenomenon is probably due to the absence, reduced density, genetic aberration or desensitization of SSTs [11–13]. Pasireotide (PAS), a novel SRL with multireceptor-binding profile, has been recently used in the therapy of acromegaly [14]. When compared with OCT, PAS has a higher binding affinity to SST_5_, SST_1_ and SST_3_ and results in rapid recycling of SST_2_ to the plasma membrane after endocytosis [15]. PAS-long-acting release (LAR) showed a better biochemical control rate than OCT or lanreotide in naïve patients with acromegaly or resistant to conventional SRLs [16–19]. Despite the clinical efficacy of PAS in acromegaly, the antitumor activity of this compound has been studied in vitro on short-term with contradictory effects.

On this basis, we evaluated the antiproliferative, antisecretory, and antiangiogenic activities of OCT and PAS in rat GH-secreting pituitary tumor cell lines (GH3 and GH4C1) after long-term incubation.

## Materials and methods

### Drug preparation and cell line cultures

OCT Acetate and PAS Pamoate were kindly provided by Novartis and diluted in DMSO at a concentration of 10^−3^ M. Rat GH-secreting pituitary tumor cell lines, GH3 and GH4C1 were provided by ATCC. GH3 cells were grown at 37 °C in F12 with Kaighn’s Modification medium, while GH4C1 in DMEM/F-12 medium, both containing 10% fetal bovine serum, 2 mM glutamine and 10^5^ U/l penicillin–streptomycin and maintained in a humidified atmosphere of 5% CO_2_. The cells were grown in 75 cm^2^ flasks and passed once every 4–7 days on a 1:2 split. They are characterized to be loosely adherent cells with floating clusters.

### RNA isolation

Total RNA was extracted from GH3 and GH4C1 cells with tryzol (Invitrogen, California, USA) according to the manufacturer’s instructions. RNA samples were stored at − 80 °C. In each reaction 500 ng of the total RNA was reverse-transcribed into complementary DNA (cDNA) with oligo(dT) primers using GoScript™ Reverse Transcription System (cat. A5000, Promega Corporation, Madison, USA) following the manufacturer’s instructions.

### Touchdown-polymerase chain reaction (TD-PCR)

TD-PCR was performed for evaluating the expression of SST_1_, SST_2,_ SST_3_, SST_4_ and SST_5_ in GH3 and GH4C1 cells. Touchdown PCR conditions for SST_1_ and SST_5_ consisted in 94 °C for 5 min, a first stage of 10 cycles consisting of a denaturation step of 94 °C for 30 s, an annealing step of 30 s that began at 65 °C and decreased by 0.5 °C per cycle until it reached 60 °C and an elongation step of 72 °C for 30 s, then a second stage of 35 cycles with an annealing temperature of 60 °C followed by a final extension of 72 °C for 7 min. For SST_2_ the first stage consisted of an annealing temperature of 65 °C (decreasing by 0.5 °C per cycle until 57 °C) for 16 cycles, followed by the second stage of 25 cycles at 57 °C of annealing. For SST_3_, the first stage consisted of an annealing temperature of 62 °C (decreasing by 0.5 °C per cycle until 54 °C) for 16 cycles, followed by the second stage of 24 cycles at 54 °C of annealing. For SST_4_, finally, the first stage consisted of 6 cycles to decrease the annealing temperature from 53 to 50 °C, while the second stage was composed of 39 cycle at 50 °C. PCR reactions were carried out in a total volume of 25 μL containing 5 µl of 5X reaction buffer with MgCl_2_, 1 µl of 10 mM of dNTPs, 1 µl of 10 pmol/µL of primer forward and reverse each, 1 µL of cDNA sample and 0.25 µl of 5 u/µL GoTaq^®^ G2 DNA Polymerase (M784B, Promega Corporation, Madison, USA). For SST_1_ and SST_4_ reaction, 10% of DMSO was also added to the volume. A reaction lacking template was used as negative control. As positive control, PCRs were conducted using genomic DNA extracted from GH3 and GH4C1 using QIAamp DNA Mini Kit (according to manufacturer’s instructions), to confirm that the reaction has been set up correctly. PCR products were visualized after 2% agarose gel electrophoresis and Midori Green Advanced (MG04, Nippon Genetics Europe) staining. The sequences of SST_1_, SST_2,_ SST_3_, SST_4_ and SST_5_ specific primers and the length of each amplified fragment were as follows: SST_1_ (expected size of 222 bp): sense, 5’-GCA AGC AGG AAA GGA GCT GCT-3’, and antisense, 5’-GCT CCA ACT GAG GCC GTC TG-3’; SST_2_ (expected size of 249 bp): sense, 5’-GTG CTC GTG GAA AAG CAA GAT GTC A-3’, and antisense, 5’-CGT GAG GAC CGC GTT GCT TGT CA-3’; SST_3_ (expected size of 256 bp): sense, 5’-CGT AAG GTT TGG GCT AGT TG-3’, and antisense, 5’-AAC CAC GTA GAT CAC CAG TG-3’; SST_4_ (expected size of 240 bp): sense, 5’-TCG TGC TAA TGG TGG TGA CT-3’, and antisense, 5’-CAG CAC CTC CAG TTG TTT CC-3’; SST_5_ (expected size of 264 bp): sense, 5’-CCC TGT CCT GCA CAG AGA CAC G-3’, and antisense, 5’-TGT CTT CAT CTT GGC GTG CCG CA-3’. A set of mouse β-actin primers was used as control (expected size of 245 bp): sense, 5’-GTG GGC CGC TCT AGA CAC CA-3’, and antisense, 5’-CGG TTG GCC TTA GGG TTC AGG GGG G-3’ [19]. All primers were obtained from Eurofins Scientific (Milan, Italy).

### Cell viability assay

GH3 and GH4C1 cells were seeded in 96 well plates at a density of 1.5 × 10^4^ cells/well. The plates were then placed in a 37 °C, 5% CO_2_ incubator. Cell culture medium of both cell lines was replaced the day after with medium containing different concentrations of OCT and PAS (ranging from 10^–11^ to 10^−4^ M) or the vehicle Dimethyl Sulfoxide (DMSO) as control (CTR) for 3 days. For the experiment of long-term incubation, the medium was replaced with a new one containing drugs or vehicle at the same different concentrations for further 3 days, at the end of which cells were analyzed by a cell viability assay, the CellTiter 96^®^ AQueous One Solution Cell Proliferation Assay (MTS, Promega, cat. G3580), according to the manufacturer’s instructions.

### Analysis of cell cycle and apoptosis by flow cytometry

GH3 and GH4C1 cells were plated in duplicates in six-well plates at a density of 1.5 × 10^5^ cells/well. The following day, cell culture medium was replaced with medium containing OCT and PAS or vehicle for 3 days as CTR. Then, the medium was replaced with a new one containing drugs or vehicle at the same different concentrations for further 3 days, at the end of which cells were harvested by gentle trypsinization, washed three times with cold phosphate-buffered saline (PBS), calcium and magnesium-free, and collected by centrifugation at 1200 × *g* for 5 min.

For cell cycle evaluation, cells were re-suspended at the concentration of 10^6^ cells/ml and directly stained with propidium iodide (PI) (Sigma-Aldrich, USA) staining solution prepared with 50 μg/ml PI, 0.6 μg/ml RNase A and 0.05% Triton X-100 in 0.1% sodium citrate and incubated at 4 °C for 30 min. For apoptosis, cells were re-suspended in 1X binding buffer (0.1 M HEPES/NaOH, pH 7.4, 1.4 M NaCl, 25 mM CaCl_2_) at a concentration of 10^6^ cells/ml and stained with 5 μl of Annexin V-FITC (BD Pharmingen, San Diego, CA, USA) plus 10 μl PI (50 μg/ml in PBS). Flow cytometric analysis was performed using FACSCalibur instrument (BD Bioscience, San Jose, CA, USA) and CellQuest software, as previously described [20].

### GH level assay

GH3 cells were plated in duplicates in six-well plates at a density of 1.5 × 10^5^ cells/well. The following day and after 24 h from the first treatment, cell culture medium was replaced with medium containing OCT and PAS or vehicle as CTR. After 24 and 48 h from the first treatment, cell culture media were collected and stored at − 80° C until analyzed. Rat GH was measured by a rat/mouse GH ELISA (EMD Millipore, Billerica, Massachusetts, cat. #EZRMGH-45K) according to the manufacturer’s procedure.

### In vivo* zebrafish assay for tumor-induced angiogenesis*

Adult zebrafish (Danio rerio) were maintained, according to European laws (2010/63/EU and 86/609/EEC). 48 h post-fertilization (hpf) *Tg(fli1a:EGFP)*^*y1*^ transgenic embryos were anesthetized with tricaine (Sigma-Aldrich) and implanted with GH-3 and GH4C1 cells, using a procedure previously described for neuroendocrine tumors [21–23]. Briefly, tumor cells were labeled with a red fluorescent viable dye (CellTrackerTM CM-DiI, Invitrogen), resuspended with PBS, and grafted into the subperidermal space of *Tg(fli1a:EGFP)*^*y1*^ embryos, close to the sub-intestinal vessels (SIV) plexus. As control of the implantation, we considered embryos injected with only PBS, the cell resuspension solution. This transplantable platform was used to test the effects of SRLs effects on tumor-induced angiogenesis. Before the implantation, tumor cells were pretreated with DMSO vehicle, as CTR, and with 2 × 10^–5^ M OCT and PAS for 6 days. After the implantation, DMSO vehicle and SRLs (10^–4^ M) were injected into the Cuvier Duct, as previously described [24]. Assays were performed 3 times, considering about 20 embryos in each experimental group. As arbitrary unit (A.U.) of tumor-induced angiogenesis. We calculated by Fiji software the total cumulative length of vessels sprouting from the plexus of subintestinal vessels (SIV) and the common cardinal vein (CCV) in each embryo at 24 and 48 h post implantation (hpi). The average ± S.E.M was statistically compared between the experimental groups with GraphPad Prism 5.0 (GraphPad Software, San Diego, CA).

### Statistical analyses

All experiments were carried out at least 3 times and gave comparable results. For statistical analysis, GraphPad Prism 5.0 (GraphPad Software, San Diego, CA) was used for cell viability assay, cell cycle and apoptosis. Half maximal effective concentration (EC_50_), as an indicator of drug potency, was calculated using nonlinear regression curve-fitting program. The comparative statistical evaluation among groups was first done by Analysis of variance (ANOVA). Statistical comparisons of the logEC50 and maximal inhibitory effect (as an indicator of drug efficacy) were performed with the extra sum-of-squares *F* test approach (cutoff at *p* = 0.05). When significant differences were found, a comparison between groups was made using the Newman-Keuls test. The unpaired Student's t test was chosen to analyze the effects of OCT and PAS on GH concentration. In all analyses, values of *p* < 0.05 were considered statistically significant. The values reported in the figures are the mean ± Standard Error of the Mean (S.E.M).

## Results

### Expression of SSTs in GH3 and GH4C1 cells

**Fig. 1:**
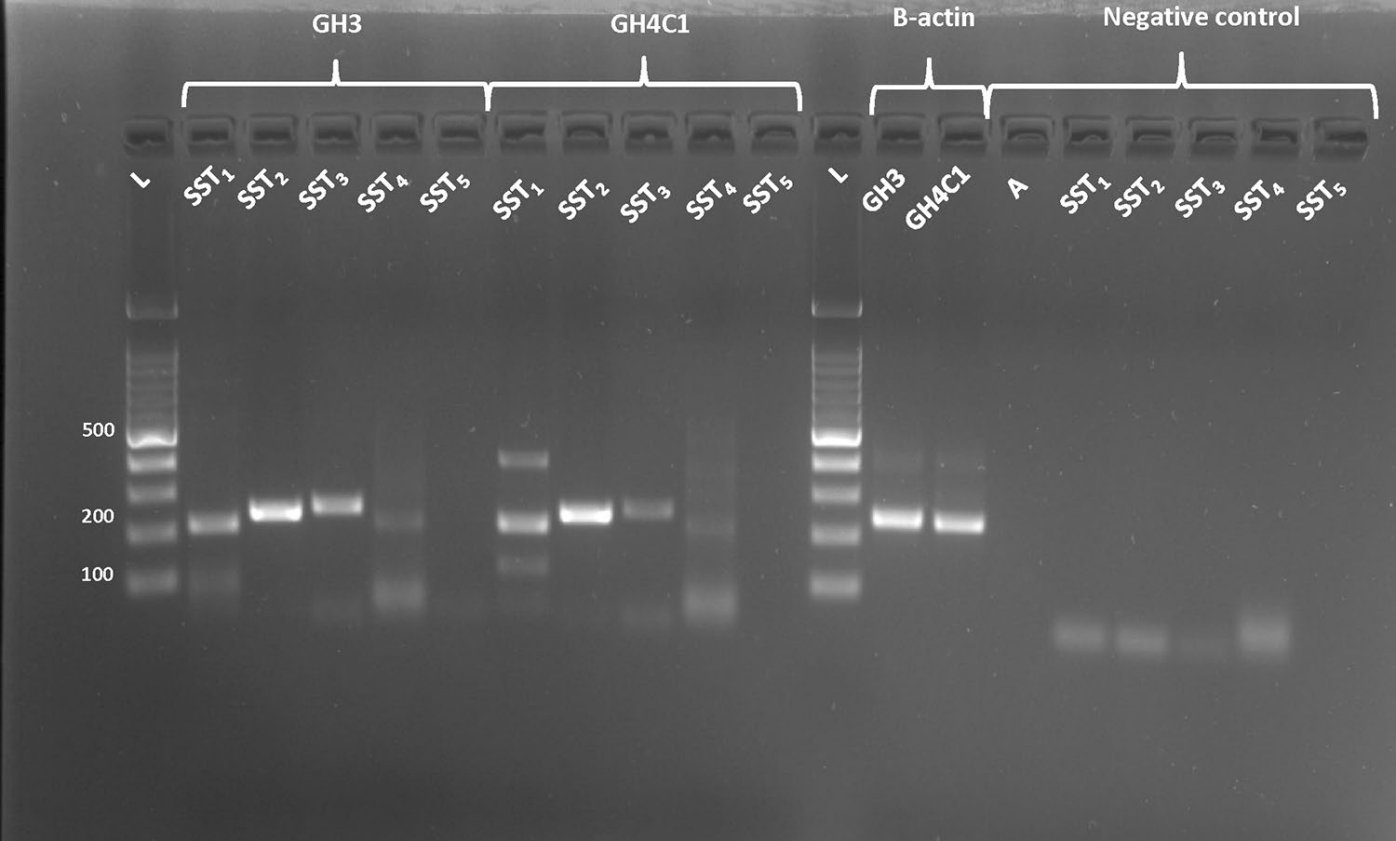
Representative results of SST_1_ (222 bp), SST_2_ (249 bp) SST_3_ (256 bp), SST_4_ (240 bp) and SST_5_ (264 bp) mRNA expression, detected by TD-PCR, in GH3 and GH4C1 cell lines. PCR reactions contained the appropriate subtype-specific primers and water as a negative control. The quality of cDNA was confirmed by polymerase chain reaction of samples with primers for β-actin (A). L: Ladder

We evaluated the mRNA expression of SST_1_, SST_2_, SST_3_, SST_4_ and SST_5_ in GH3 and GH4C1 cells by TD-PCR (Fig. 1). In both cell lines, we observed a strong expression of SST_2_, a moderate expression of SST_1_ and SST_3_ and a very weak expression of SST_4_ subtype transcript, while SST_5_ was not detected.

### Long-term SRLs treatment decreased viability of rat GH-secreting pituitary tumor cell lines

**Fig. 2:**
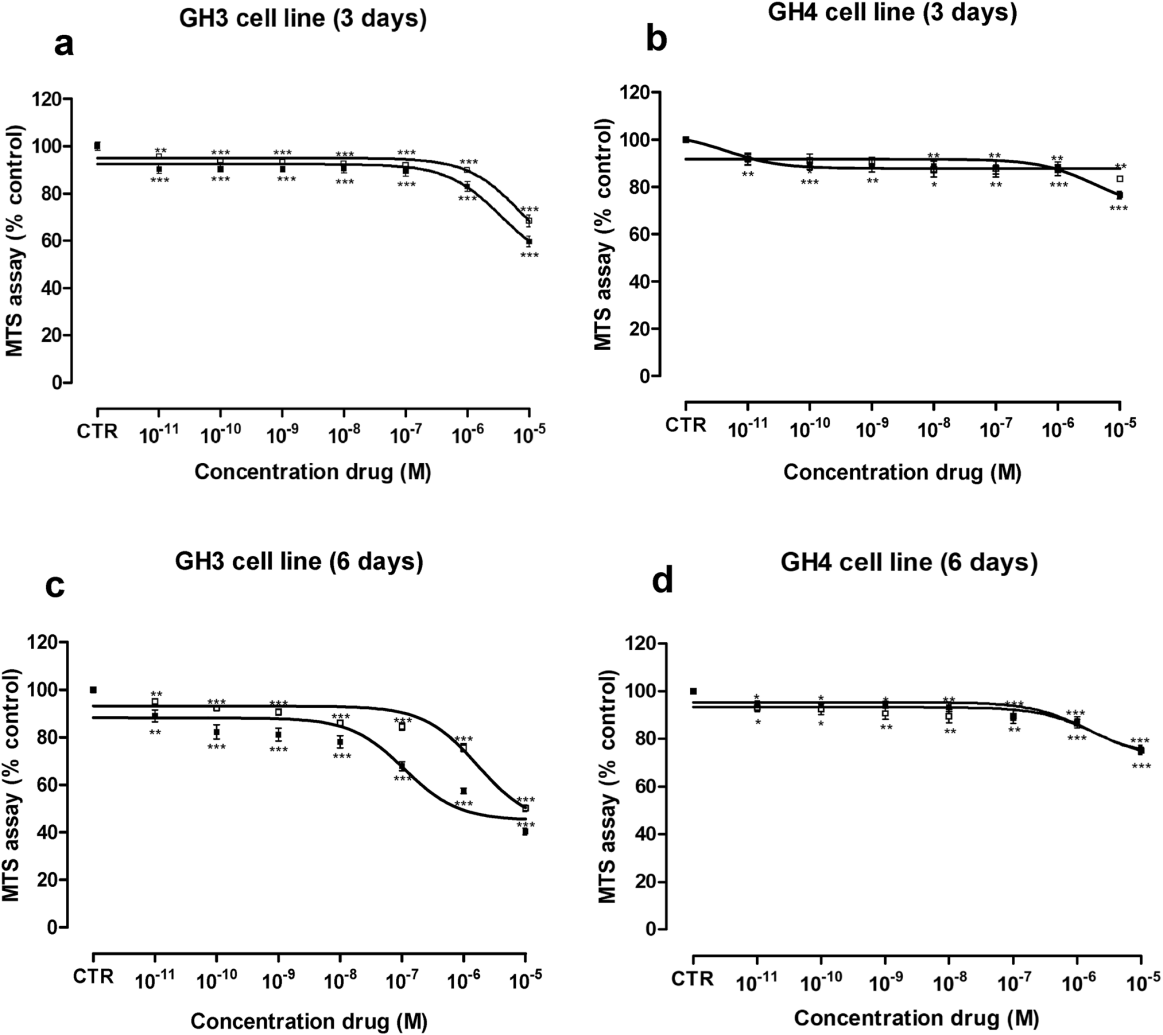
Effects of OCT (□) and PAS (■) on viability of GH3 (**a**, **c**) and GH4C1 (**b**, **d**) cell lines, as measured by MTS assay. Cells were incubated for 3 (**a**, **b**) and 6 days (**c**, **d**) without or with the drug at different concentrations (range 10^–11^–10^–5^ M). Dose–response curves represented best fit values of nonlinear regression (curve fit) of log (concentration drug) versus the percentage of vehicle-treated control (CTR). Values represent the mean and S.E.M. of at least three independent experiments in six replicates. *p < 0.05; **p < 0.01; ***p < 0.001 vs CTR

Dose–response curves showed that both OCT and PAS significantly inhibited the viability of GH3 and GH4C1 cells in a dose-dependent manner (Fig. 2).

In GH3 cells, we observed comparable anti-tumor activity between OCT (EC_50_: 7.5 × 10^–6^ M, maximal inhibition: − 52%) and PAS (EC_50_: 3.4 × 10^–6^ M, maximal inhibition: − 51%) after 3 days of incubation. Indeed, no significant differences between both drugs for EC_50_ and maximal inhibition values have been found (Fig. 2a). After 6 days of incubation (Fig. 2c) a more potent inhibitory activity has been observed with PAS compared to OCT (EC_50_: 1.1 × 10^–7^ M, EC_50_: 1.7 × 10^–6^ M, respectively, *p* < 0.0001), while a comparable efficacy has been found between PAS and OCT (maximal inhibition: − 55%, maximal inhibition: − 57%, respectively).

In GH4C1 cells, mild and comparable inhibitory effects on cell viability have been observed with both drugs after 3 days (Fig. 2b, OCT EC_50_: 4.5 × 10^–12^ M, maximal inhibition: − 12.2%; PAS EC_50_: 3.7 × 10^–6^ M, maximal inhibition: − 29%) and 6 days (Fig. 2d, OCT EC_50_: 1.881 × 10^–6^ M, maximal inhibition: -28%; PAS EC_50_: 1.4 × 10^–6^ M, maximal inhibition: − 27%) of incubation. Indeed, no significant differences have been observed between EC_50_ and the maximal inhibitory effect of both drugs. For further experiments, we have selected the EC_50_ concentrations of OCT and PAS after 6 days of incubation.

### Long-term effect of SRLs on cell cycle phases of rat GH-secreting pituitary tumor cell lines

**Fig. 3:**
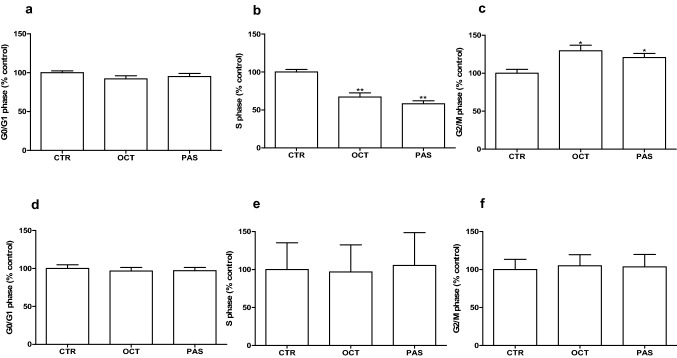
Cell cycle analysis after 6 days of incubation with OCT, PAS in GH3 (**a**–**c**) and GH4C1 (**d**–**f**) cell lines. Cells were detected by FACS analysis after staining with propidium iodide. Vehicle-treated control (CTR) values have been set to 100%. *p < 0.05; **p < 0.01 vs CTR

After 6 days of incubation both drugs significantly decreased the percentage of GH3 cells in S phase, (OCT: − 33%, vs control, *p* < 0.01; PAS: − 42%, vs control, *p* < 0.01) and increased the number of cells in G_2_/M phase (OCT: + 30%, vs control, *p* < 0.05; PAS: + 21%, vs control, *p* < 0.05) (Fig. 3a–c). No statistically significant effect on cell cycle distribution was observed after incubation with both SRLs in GH4C1 cells (Fig. 3d–f).

### Long-term effect of SRLs on apoptosis of rat GH-secreting pituitary tumor cell lines

**Fig. 4:**
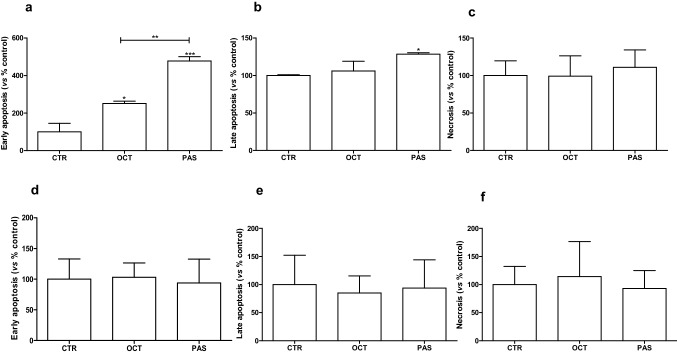
Modulation of cell death analysis after 6 days of incubation with OCT and PAS in GH3 (**a**–**c**) and GH4C1 (**d**–**f**) cell lines through flow cytometry with Annexin V and propidium iodine. The proportions of early (**a**, **d**), late (**b**, **e**) apoptotic, necrotic (**c**, **f**) cells are expressed as percentage compared with vehicle-treated control (CTR). Values represent the mean and SEM of at least three independent experiments. *p < 0.05; **p < 0.01; ***p < 0.001

OCT induced a statistically significant increase of GH3 cells in early apoptosis (+ 151% vs untreated cells, *p* < 0.05) (Fig. 4a). PAS significantly induced a prominent increase of GH3 cells in both early (+ 378% vs untreated cells, *p* < 0.01) and late apoptosis phase (+ 28% vs untreated cells, *p* < 0.05) after 6 days of incubation (Fig. 4a, b). Both treatments did not significantly affect necrosis (Fig. 4c). In GH4C1 cells both drugs did not significantly modify the fractions of cells in early apoptosis, late apoptosis, and necrosis compared to controls (Fig. 4d–f).

### Modulation of GH release after SRLs exposure

**Fig. 5:**
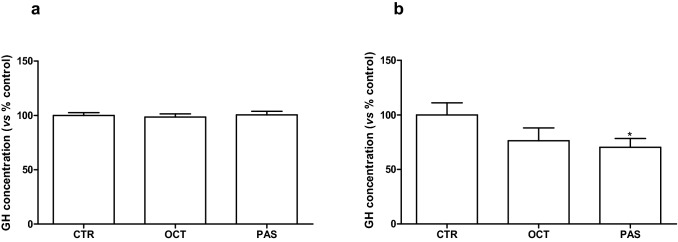
Effect of SRLs on GH secretion in GH3 cell line. GH was measured by a rat/mouse GH ELISA (EMD Millipore, Billerica, Massachusetts) on cell culture media after 24 h (**a**) and 48 h (**b**) of incubation. GH values were normalized to the cellular proteins of each group. Results were expressed as a percentage compared with the vehicle-treated control (CTR) and represent the mean and SEM of at least three independent experiments. *p < 0.05 vs CTR

We evaluated the antisecretory activity of OCT and PAS. In GH3 cells, no GH release modulation was observed after 24 h of exposure with both SRLs (Fig. 5a). After 48 h of incubation, only PAS significantly inhibited GH secretion (− 30%, compared to untreated cells, *p* < 0.05) (Fig. 5b), while OCT resulted in a mild and not significant inhibition in GH secretion.

### SRLs effect on GH3 cell line-induced angiogenesis

**Fig. 6:**
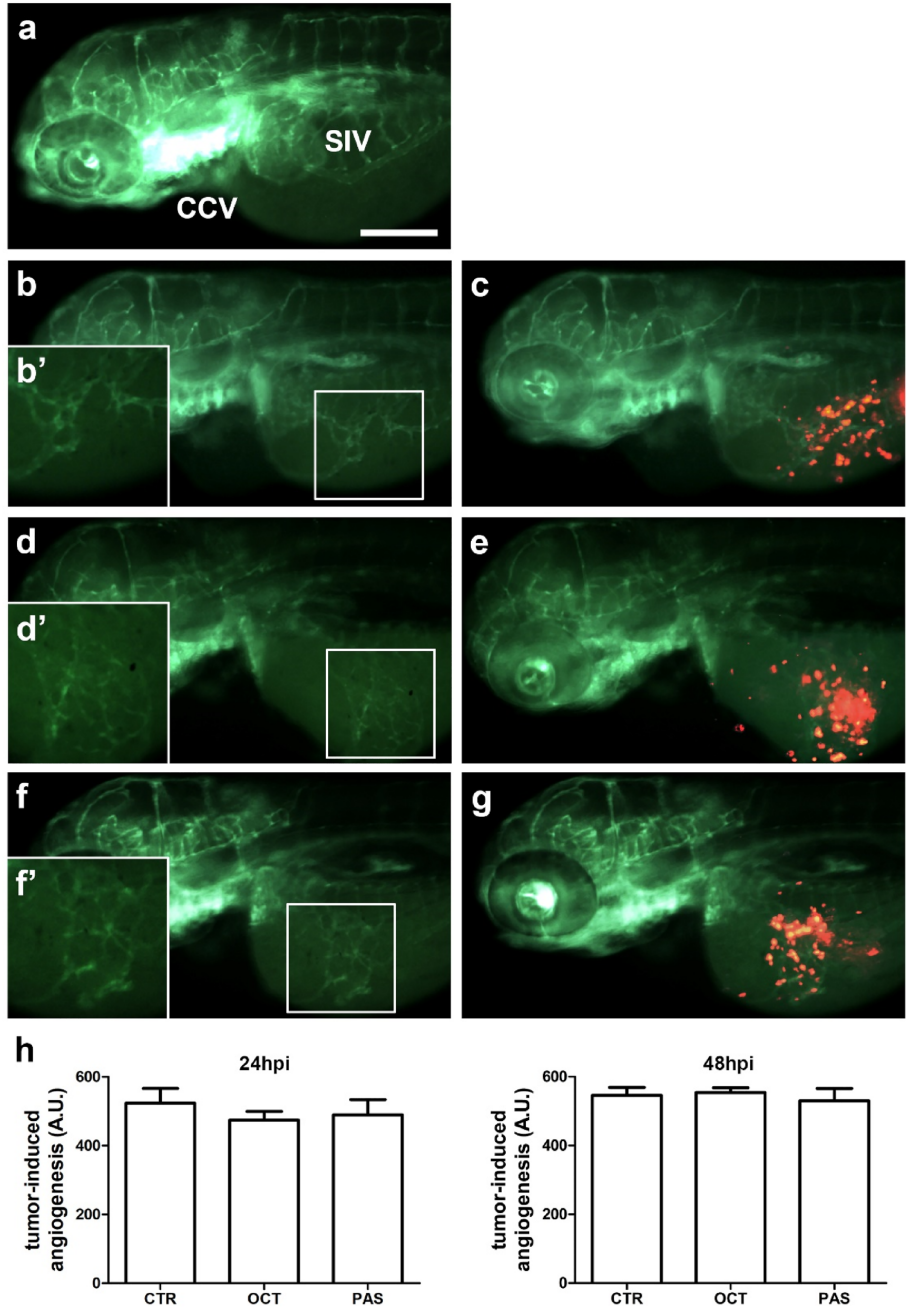
Effect of treatment with SRLs on GH-3 cells-induced angiogenesis. Representative epifluorescence images of 48 hpi *Tg(fli1:EGFP)*^*y1*^ zebrafish embryos injected with only PBS (**a**) or implanted with GH3 cells (**b**-**g**) and subsequently treated with DMSO vehicle (**b** and **c**), OCT (**d** and **e**) and PAS (**f** and **g**). The red channel was omitted in panels **b**, **b**′, **d**, **d**′, **f** and **f**′ to highlight the tumor-induced microvascular network. Digital magnifications of graft region are shown in white boxed regions **b**′, **d**′ and **f**′. The peritumoral density of endothelial structures, that sprouted from the SIV and CCV and reached the GH-3 tumor mass, did not result in difference in SRL-treated embryos compared to CTR. Here we show the quantification of tumor-induced endothelial structures at both 24 and 48 hpi (**h**). All images are oriented so that rostral is to the left and dorsal is at the top. Scale bar in a, 100 µm

**Fig. 7:**
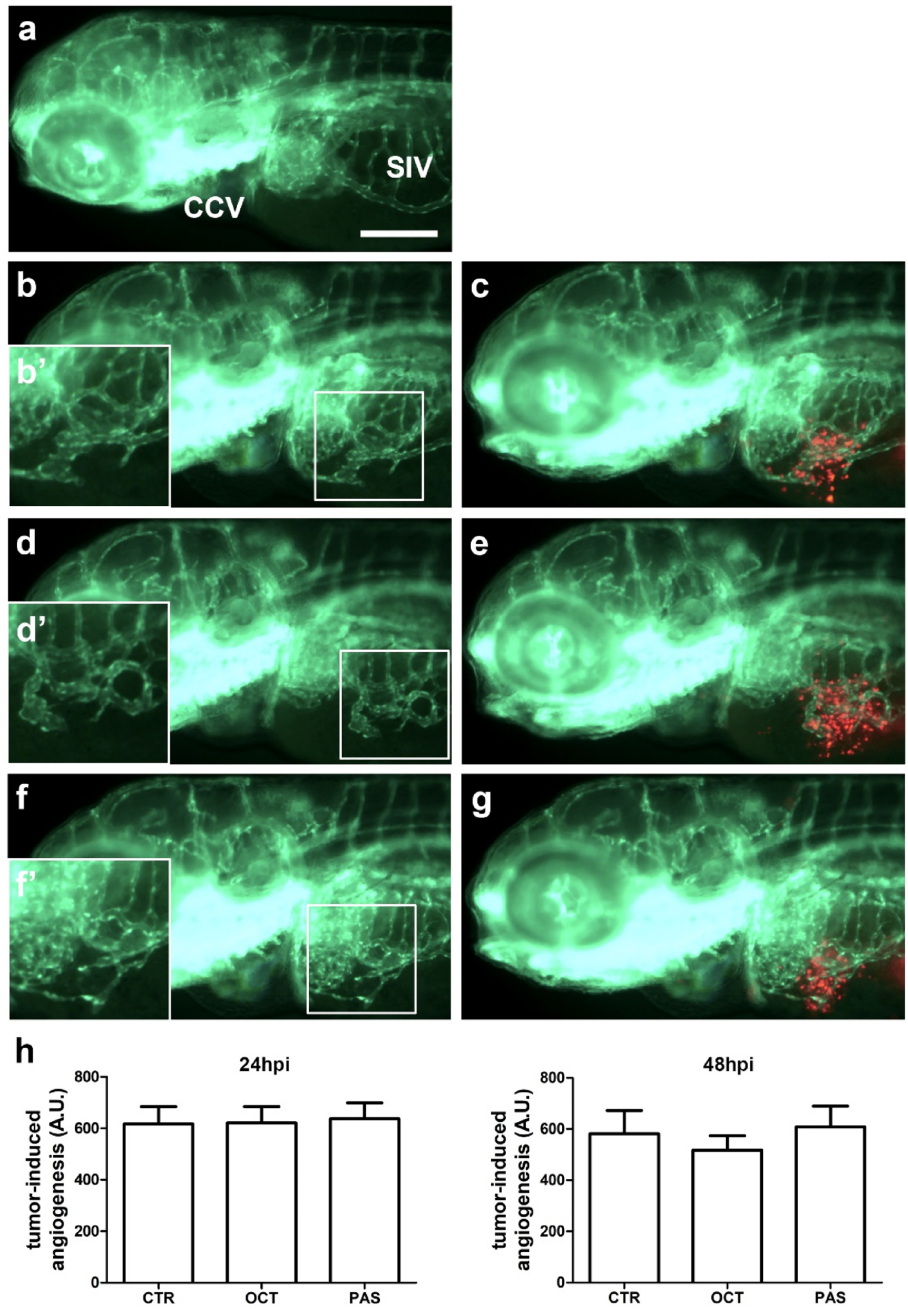
Effect of treatment with SRLs on GH4C1 cells-induced angiogenesis. Representative epifluorescence images of 48 hpi *Tg(fli1:EGFP)*^*y1*^ zebrafish embryos injected with only PBS (**a**) or implanted with GH4C1 cells (**b**–**g**) and subsequently treated with DMSO vehicle (**b** and **c**), OCT (**d** and **e**) and PAS (**f** and **g**). The red channel was omitted in panels **b**, **b**′, **d**, **d**′, **f** and **f**′ to highlight the tumor-induced microvascular network. Digital magnifications of graft region are showed in white boxed regions **b**′, **d**′ and **f**′. The treatment with SRLs did not reduce the network density of endothelial structures, that sprouted from the SIV and CCV and reached the GH4C1 tumor mass, compared to vehicle-treated CTR embryos. Here we show the quantification of tumor-induced endothelial structures at both 24 and 48 hpi (**h**). All images are oriented so that the rostral is to the left and dorsal is at the top. Scale bar in a, 100 µm

To analyze the antiangiogenic potential of OCT and PAS on GH3 and GH4C1 cell lines, we used an innovative in vivo platform, that we have recently developed implanting neuroendocrine tumors cells in *Tg(fli1:EGFP)*^*y1*^ zebrafish embryos [22]. Before the implantation, GH3 or GH4C1 cells were pre-treated in vitro with DMSO (CTR), OCT and PAS for 6 days. These cells were then implanted in 48 h post fertilization (hpf) *Tg(fli1:EGFP)*^*y1*^ embryos into the subperidermal space. After the implantation, DMSO, OCT and PAS were injected into the Cuvier duct. Afterwards, we evaluated the density of tumor-induced endothelial structures around the tumor graft. In our in vivo assays, we did not observe any significant change of tumor-induced angiogenesis after the treatment with OCT and PAS in a temporary window of 24 and 48 hpi for GH3 (Fig. 6) and GH4C1 (Fig. 7) cells.

## Discussion

This study evaluated the long-term effects of different SRLs on GH-secreting pituitary tumor cell lines, supporting a more potent anti-tumor effect of PAS than OCT.

SSTs, especially SST_2_ and SST_5_, are the main classic targets to inhibit excessive hormone release and cell growth in GH secreting pituitary tumors [25]. The anti-proliferative effects of SRLs in tumors are directly exerted through the induction of apoptosis and cell cycle inhibition, and indirectly through inhibition of angiogenesis and secretion of several growth factors [26]. Although several clinical trials revealed that PAS has a superior efficacy over OCT in patients with acromegaly [17–19], there are several contradictory data concerning the antitumor activity and related mechanisms [27–29]. In addition, most of the in vitro studies are related to a short-term incubation of GH-secreting pituitary tumor cells with SRLs.

OCT (10^–8^ M) exerted a significant, but transient, inhibition of GH3 cell growth with a maximum effect at 24 h, no longer detectable after 48 h [27]. Hubina and coworkers demonstrated that both OCT and PAS decreased GH3 cell proliferation after 72 h incubation time through inhibition of ERK-pathway and an increase in p27 expression at 10 min of exposure [28]. Both SRLs (10^–8^ M) showed in vitro comparable inhibition of cell viability after incubation for 24–72 h in primary GH-secreting pituitary tumor cells [29]. These discrepancies between clinical trials and in vitro studies are probably related to differences in both receptor expression pattern and activity of SSTs after interactions with SRLs [30]. The expression of these receptors has been already described in rat GH-secreting pituitary tumor cell lines. SST_1_ and SST_2_ were the most expressed subtypes in native GH3 cells [31–34]. Wild-type GH4C1 showed mRNA abundance for SST_1_, SST_2_, SST_3_ [30, 35]. The high SST_2_ expression in rat GH3 cells [31] may explain the receptor desensitization after stimulation [36]. Indeed, PAS modulates SSTs trafficking in a clearly distinct manner from OCT. Lesche and coworkers reported that PAS caused a significantly lower internalization and rapidly recycling to the plasma membrane of SST_2_ compared to OCT after endocytosis in HEK 293 cells [15]. Indeed, PAS stimulated only phosphorylation of Ser341 and Ser343 residues of human SST_2_, which is followed by a partial receptor internalization compared to OCT [15, 37]. Another study confirmed that the degree of SST_2_ internalization by PAS was smaller compared to OCT [38]. In human pancreatic neuroendocrine tumor primary cultures PAS resulted in a rapid and transient internalization of SST_2_ followed by persistent recycling of the receptor at the cell surface [39]. While, in GH4C1 cells it has been recently observed that both OCT and PAS (10^−8^ M) resulted in a robust internalization of SST_2_ and a comparable inhibition of cell proliferation after 48 h [40]. Therefore, a cell and tissue type variability of SST functions and intracellular trafficking may have a role to explain such divergent responses in several studies.

In vitro experiments with long-term incubation should better evaluate the antitumor activity of SRLs. Indeed, this experimental condition is closer to the clinical reality. In the current work, we found only a mild and comparable inhibition of cell viability in GH3 and GH4C1 cells after 3 days of incubation with OCT or PAS and in GH4C1 cells after 6 days. While, in GH3 cells the antitumor activity of PAS was more potent than that of OCT after 6 days. These data were also confirmed after 9 days of incubation (data not shown). We observed a similar SSTs profile in both cell lines, with a strong expression of SST_2_, a moderate expression of SST_1_ and SST_3_ and a very weak expression of SST_4_ subtype transcript. Therefore, we cannot exclude that the differences in the inhibitory effects of SRLs observed between GH3 and GH4C1 cells are probably due to different post-receptor mechanisms. While the stronger inhibition of cell viability observed after 6 days with PAS than OCT in GH3 cells could be related to the differential SST downregulation stimulated by the two drugs. However, to our knowledge, there are no data currently reporting a differential modulation of SST_2_ expression after long-term treatment with SRLs.

Direct antitumor effects of SRLs are modulated by the induction of cell cycle lock and apoptosis [41]. It has been already demonstrated that in GH3 cells, OCT had a cytostatic effect by blocking cells in G_0_/G_1_ phase after 24 h of incubation [42], through the inhibition of the early response gene c-fos or DNA binding of the heterodimeric transcription factor complex [43]. However, unless OCT was replenished, cell cycle block was transient and overcome by 36–48 h [42]. In addition, both somatostatin-14 and OCT were unable to induce apoptosis in GH3 cells after short-term incubation [42]. On the light of this experimental background, modulation of cell cycle and apoptosis after PAS and after a long-term treatment with SRLs has not been exhaustively documented in GH-secreting tumor cells. After 6 days of incubation, only in GH3 cells, we found that both OCT and PAS induced a comparable decrease of cells in S phase and an increase in G_2_/M phase. Interestingly, after a long-term incubation both SRLs induced apoptosis in only GH3 cells, with a more potent proapoptotic activity after PAS compared to OCT.

The anti-proliferative effects are independent of anti-secretory actions of SRLs both in vivo and in vitro [44, 45]. Indeed, each SST can have a different effect on the modulation of cell proliferation and GH secretion [46]. OCT (10^–6^ and 10^–7^ M) reduced GH production after 24 h of incubation of GH3 cell line stimulated by forskolin [47] and after 72 h (10^–8^ and 10^–7^ M) [48]. GH suppression by OCT (10^–8^ M) ranged from 8.5 to 73.7% in GH-secreting primary cells of 24 pituitary tumors from acromegalic patients after 72 h of treatment [49]. A recent critical analysis of preclinical studies comparing the antisecretory activity of PAS vs OCT in somatotroph tumor primary cultures, showed comparable inhibitory effects on GH secretion (incubation time from 4 to 72 h) [50]. An in vitro long-term study on human primary GH secreting pituitary tumor cells found a dose-dependent inhibition of GH release after incubation with OCT for periods ranging from 4 days up to 3 weeks, and a parallel increase in the intracellular GH levels and GH mRNA expression [51]. Due to the low GH production of GH4C1 cells, we evaluated the effects of OCT and PAS on GH release in only GH3 cells conditioned media. For these experiments, we selected a short incubation time, in order to avoid any interference on GH concentrations related to the antiproliferative activity of SRLs. We found a significant decrease in GH secretion after 48 h of incubation only with PAS. At this time, we did not observe any effect on the viability of GH3 cells after PAS or OCT.

Somatostatin and its analogs are also able of inhibiting angiogenesis. SST_1_ is highly expressed in vessels, where it inhibits endothelial proliferation, migration, and neovascularization [52]. OCT (10^–10^–10^–6^ M) and PAS (10^–9^–10^–6^ M) inhibited proliferation of HUVECs, preferentially expressing SST_2_ and SST_5_ during proliferation, in a dose-dependent manner [53]. SST_3_ has been shown to downregulate the transcription of vascular endothelial growth factor (VEGF), which drives the development of new vessels in the growing tumor during hypoxia. The inhibition of endothelial nitric oxide synthase by SST_1_, SST_2_ and SST_3_ may contribute to the anti-angiogenic activity of SRLs [54]. Vidal and coworkers showed a lower microvascular density in GH-producing tumors treated with OCT than those untreated, although the differences did not reach statistical significance [55]. However, the role of SRLs in modulating tumor-induced angiogenesis is poorly understood. We have recently developed an innovative angiogenesis assay based on the injection of human neuroendocrine tumor cells in transgenic zebrafish embryos [22]. Inoculation of tumor cells in zebrafish embryos can induce a potent angiogenic response through the secretion of several growth factors [22]. VEGF/fibroblast growth factor (FGF) gradient produced by the tumor is able to guide the sprouting of new blood vessels from the close vascular network (SIV and CCV). In our model, implantation of GH3 and GH41C cells in zebrafish embryo significantly stimulated angiogenesis within 24–48 h from engraftment, while long-term pre-incubation with OCT or PAS showed no significant effect on the migration and growth of sprouting vessels toward both tumor implants.

The main limitation of this study is the use of only two cell lines. However, only a few preclinical models of acromegaly are available. GH3 and GH4C1 represent the most widely used GH-secreting pituitary tumor cell lines for the studies of the somatostatin network.

In conclusion, we found that a long-term incubation of GH3 cells with PAS showed a more potent antitumor activity compared to that reported after OCT, while no significant impact has been observed on tumor-induced angiogenesis. This effect is modulated by a cell cycle perturbation and a relevant pro-apoptotic activity.
